# Planetary health literacy: A conceptual model

**DOI:** 10.3389/fpubh.2022.980779

**Published:** 2023-01-16

**Authors:** Carmen Jochem, Julia von Sommoggy, Anna-Katharina Hornidge, Eva-Maria Schwienhorst-Stich, Christian Apfelbacher

**Affiliations:** ^1^Department of Epidemiology and Preventive Medicine, University of Regensburg, Regensburg, Germany; ^2^Department of Epidemiology and Preventive Medicine/Medical Sociology, University of Regensburg, Regensburg, Germany; ^3^German Institute of Development and Sustainability, Bonn, Germany; ^4^Department for Political Sciences and Sociology, University of Bonn, Bonn, Germany; ^5^Department of General Practice/Family Medicine, University Hospital Würzburg, Würzburg, Germany; ^6^Teaching Clinic of the Faculty of Medicine and Institute of Medical Teaching and Medical Education Research, University of Würzburg, Würzburg, Germany; ^7^Institute of Social Medicine and Health Systems Research, Otto-von-Guericke University Magdeburg, Magdeburg, Germany; ^8^Lee Kong Chian School of Medicine, Clinical Sciences Building, Nanyang Technological University Singapore, Singapore, Singapore

**Keywords:** planetary health, education, literacy, model, sustainability

## Abstract

Education for planetary health could be one of the key levers of the much-needed civilizational turn toward a sustainable and healthy future. Education goes beyond information provision and passing on of knowledge and includes competencies to transfer knowledge from one decision situation to another. There are a range of different literacy concepts from various research perspectives that aim to improve such competencies. While many contain aspects highly relevant for planetary health, there is still no comprehensive and integrative planetary health approach. To fill this research gap, we present a conceptual model of planetary health literacy. By zooming into the model, further details on the necessary core competencies of accessing, understanding, appraising, and applying information in order to make judgements and take decisions regarding planetary health can be found. Zooming out of the model allows a holistic planetary health perspective and shows the potential and opportunities of planetary health literacy for the health of humans and ecosystems. Planetary health literacy encompasses both a life-course and a transgenerational approach, at the individual, societal, and global level. Future educational programs focusing on planetary health could integrate the conceptual model to increase planetary health literacy of individuals, including relevant health literacy agents, and of societies.

## 1. Introduction

There is no doubt that humankind is facing a planetary crisis of enormous dimensions. Climate change, biodiversity loss, and increasing pollution are clear signs of the planetary state of emergency and have both direct and indirect negative effects on human health (e.g., by increasing the frequency of climate and weather extremes and by reducing food and water security) (1–4). Although human health has improved in recent decades (in terms of life expectancy etc.), humanity is far from being healthy. “Planetary health” has been coined to denote “the health of human civilization and the state of the natural systems on which it depends” (5).

On a global scale, the increasing burden of non-communicable diseases reflect the detrimental effects of social and commercial determinants of ill health and associated unhealthy lifestyles (6, 7). In addition, infectious diseases, as well as maternal and child mortality still represent a large burden of disease, especially in poorer population groups (6, 8).

Education for planetary health (including all levels of education, i.e., primary, secondary, and tertiary education), may be one of the key levers for the much-needed civilizational turn toward planetary health (9). By raising both individual and social awareness regarding the ways of thinking, deciding, and acting—as humans being part of our unique planet Earth—planetary health education may lead to individual and collective behavior patterns that enable lifestyles for global sustainable development.

Education provides the basis for skills like reading, writing, or calculating literacy. However, education goes beyond the sole information provision, teaching and acquiring of knowledge and includes socializing lifestyles built around reason and rationale as well as enabling individuals to develop and trust their intellectual competences and practical skillsets. A large number of different literacy concepts from various research perspectives exist and target these competences. Many of them contain aspects that are highly relevant for planetary health such as transformative literacy (10) (see Table 1). However, most of them (e.g., health literacy, environmental literacy, eco-/ecological literacy, sustainability literacy) remain within their inherent perspectives and disciplines (11–15). Some concepts (e.g., climate and health literacy, environmental health literacy) reflect interdisciplinary approaches linking environmental with health perspectives (16, 17). However, a comprehensive and integrative planetary health approach that includes all aspects relevant for planetary health is still lacking.

**Table 1:** Definitions of selected literacy concepts of relevance for planetary health.

**Health literacy**
“[…] the knowledge, motivation and competencies of accessing, understanding, appraising, and applying health-related information within the healthcare, disease prevention and health promotion setting.” (11)
**Eco-/ecological literacy**
“The ecoliterate person has the knowledge necessary to comprehend interrelatedness and an attitude of care or stewardship. […] Knowing, caring, and practical competence constitute the basis of ecological literacy. Ecological literacy, further, implies a broad understanding of how people and societies relate to each other and to natural systems, and how they might do so sustainably. It presumes both an awareness of the interrelatedness of life and knowledge of how the world works as a physical system.” (13)
“People who are ecoliterate cultivate compassion toward other forms of life. […]. This ability to feel empathy often stems from a deep understanding that humans are part of a broader community that includes all living beings. […] Ecoliterate people collectively practice a way of life that fulfills the needs of the present generation while simultaneously supporting nature's inherent ability to sustain life into the future.” (14)
**Environmental literacy**
“[…] is knowledge of environmental concepts and issues; the attitudinal dispositions, motivation, cognitive abilities, and skills, and the confidence and appropriate behaviors to apply such knowledge in order to make effective decisions in a range of environmental contexts. Individuals demonstrating degrees of environmental literacy are willing to act on goals that improve well-being of other individuals, societies, and the global environment, and are able to participate in civic life.” (15)
**Sustainability literacy**
“The knowledge, skills and mindsets that allow individuals to become deeply committed to building a sustainable future and assisting in making informed and effective decisions to this end.” (12)
**Transformative literacy**
“The ability to perceive, interpret and utilize information about societal transformation processes in a way that enables people to get actively involved in shaping these processes.” (10)
**Climate and health literacy**
“[…] the degree to which an individual understands the complex relationship between climate change and human health; a climate health-literate individual can recognize direct and indirect linkages between climate change and health, communicate risks, assess data, comprehend uncertainty, and make informed and responsible personal decisions or advocate for broader policies that protect health.” (16)
**Environmental health literacy**
“Environmental health literacy integrates concepts from both environmental literacy and health literacy to develop the wide range of skills and competencies that people need in order to seek out, comprehend, evaluate, and use environmental health information to make informed choices, reduce health risks, improve quality of life and protect the environment.” (17)”

In order to address this research gap, we propose a conceptual model of planetary health literacy that may contribute to a more holistic education for planetary health. The concept emphasizes the need to consider the impact of personal and collective decisions, activities, and behavior on the state of the Earth's natural systems in order to preserve it, given that human health is part of planetary health. This conceptual model may support the development of future research questions and practical interventions aimed at improving planetary health literacy within individuals and societies. The model aims at leveraging the synergies that result from planetary health literate individuals and societies to enable better individual behavioral choices and necessary structural changes for healthy people on a healthy planet.

## 2. A conceptual model of planetary health literacy

The conceptual model of planetary health literacy we propose, comprises several parts of existing literacy concepts of systemic and community- and society-oriented literacy approaches such as ecological literacy, ecoliteracy, and transformative literacy (10, 13, 14), as well as parts of an integrated model of health literacy (11). The model development process is based on the inter- and transdisciplinary cooperation of the team of authors with different disciplines from social and natural sciences and humanities combining professions and backgrounds from medicine, global health policy, international health, health sciences, public health, health promotion, health literacy, comparative ethnology, sociology, global sustainable development, (medical) education, and philosophy. By moving beyond discipline-specific approaches in creative discussions and collaborative writing processes, the team of authors brought together their professional expertise and jointly created the new conceptual model of planetary health literacy. Zooming into the conceptual model provides further details on planetary health literacy (e.g., on knowledge, core competencies, information, and knowledge environments regarding planetary health) at the individual level. The model enables a life course and transgenerational approach at the individual, societal, and global level, and it should be considered as a goal of education for planetary health. Zooming out of the health literacy model allows a holistic planetary health perspective and shows the potential and the opportunities of planetary health literacy with respect to wellbeing societies, health promotion and sustainability (18). By zooming in and out of the model, we provide an approach that is helpful for the examination of research questions at multiple scales and was already used in similar contexts (19). Following the description of the model, we will illustrate the application of planetary health literacy for several thematic areas, considering potential barriers and challenges. Table 2 shows a glossary of terms that are of relevance for the conceptual model of planetary health literacy.

**Table 2:** Glossary of terms that are of relevance for the planetary health literacy model.

**Co-benefits**
“Co-benefits are mutually positive outcomes for health and other sectors within governments, organizations and communities. Co-benefits across sectors and society at large can be achieved when health considerations are transparently taken into account in policy-making, resource allocation and service delivery.” (25)
**Commercial determinants of health**
“Activities of the private sector – including strategies and approaches used to promote products and choices – that affect the health of populations.” (25)
**Ecosystem health**
“A metaphor used to describe the condition of an ecosystem, by analogy with human health. Note that there is no universally accepted benchmark for a healthy ecosystem. Rather, the apparent health status of an ecosystem is judged on the ecosystem's resilience to change, with details depending upon which metrics (such as species richness and abundance) are employed in judging it and which societal aspirations are driving the assessment.” (41)
**Global health**
“Achieving health equity at a global level by addressing transnational health issues, determinants, and the interventions and formal structures that are beyond the control of national institutions.” (25)
**Life course**
“A culturally defined sequence of stages that people typically pass through as they progress from birth to death. Health across the lifespan reflects a complex interplay of biological, behavioral, psychological, and social protective and risk factors that contribute to health outcomes across the span of a person's life.” (25)
**Planetary health**
“The achievement of the highest attainable standard of health, well-being and equity worldwide through judicious attention to the human systems – political, economic and social – that shape the future of humanity, and the Earth's natural systems that define the safe environmental limits within which humanity can flourish.” (25)
**Public health**
“An organized activity of society to promote, protect, improve, and – when necessary – restore the health of individuals, specified groups, or the entire population. It is a combination of sciences, skills and values that function through collective societal activities and involve programmes, services and institutions aimed at protecting and improving the health of all people.” (25)
**Well-being**
“Well-being is a positive state experienced by individuals and societies. Similar to health, it is a resource for daily life and is determined by social, economic and environmental conditions.” (25)

### 2.1. Zoom in: Knowledge regarding planetary health and core competencies for planetary health literacy

**Figure 1:**
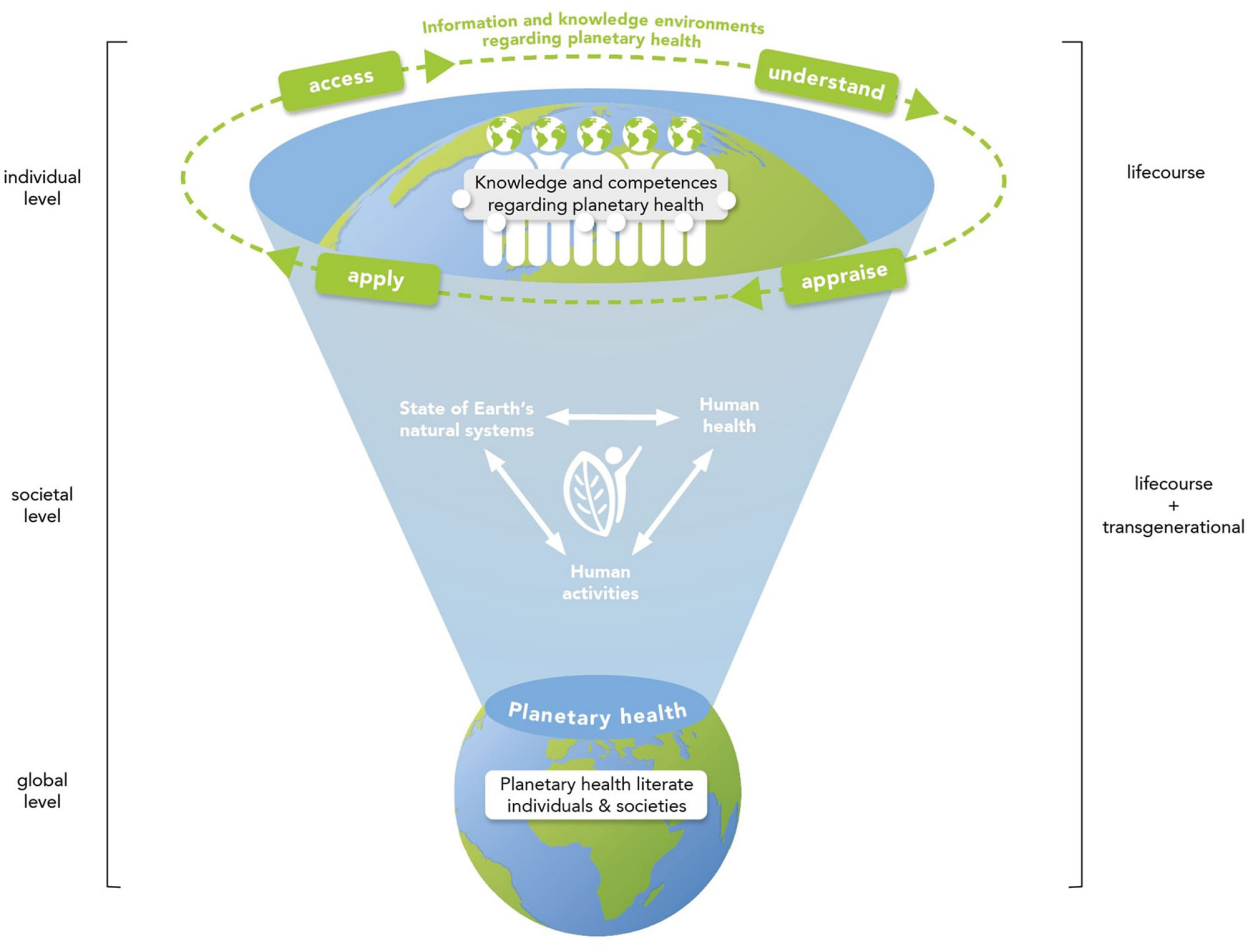
A conceptual model of planetary health literacy. Zooming into the core of the model shows the relevance of knowledge and competences regarding planetary health, i.e., regarding the interconnectedness of human health and wellbeing with the state of the natural systems, at the individual level. Zooming into the individual level—as if looking through a magnifying glass—allows highlighting the knowledge and competencies that enable individuals to access, understand, appraise and apply information and knowledge environments regarding planetary health. Zooming out of the core of the model allows a more holistic view on the planetary level and shows planetary health literate individuals and societies as the desired outcome of education for planetary health.

Zooming into the core of the model—as if looking through a magnifying glass—highlights the relevance of knowledge regarding planetary health at the individual level. The conceptual model is based on the definition of planetary health as “the achievement of the highest attainable standard of health, wellbeing, and equity worldwide through judicious attention to the human systems – political, economic, and social – that shape the future of humanity and the Earth's natural systems that define the safe environmental limits within which humanity can flourish. Put simply, planetary health is the health of human civilization and the state of the natural systems on which it depends (5)”. According to this definition, knowledge and expertise regarding planetary health refer to “the health of human civilization and the state of the natural systems on which it depends” (5) and the interconnectedness of both. It includes an understanding of the direct and indirect effects of environmental changes in the state of natural systems and ecosystem impairment such as climate change, land degradation, biodiversity loss, or urbanization on human health (Figure 1). Further, it includes knowledge on and understanding of the effects of human activities and lifestyles on both human health and health of natural systems—and the benefits to both that arise from health-promoting and ecosystem-friendly, sustainable decisions, activities and lifestyles. Importantly, knowledge and expertise on planetary health is not restricted to climate change and its effects on human health, but rather comprises all areas of nature-society interactions. Thus, it is diverse, focuses on the integratedness of these systems and has to be actively fostered in systems of knowledge production and sharing. These systems allow for this diversity bound together in overlapping subsystems, all part of one large system, our planet Earth, to be captured (17). Furthermore, in the context of planetary health, no one form of knowledge can or should be privileged over another. Rather, knowledge on planetary can be enhanced by relationship-based insights, practices and mental models from diverse knowledge systems including, for example, the valuable insights and leaderships provided by Indigenous People (20, 21).

Acknowledging the existence of other widely recognized models of health literacy (22–24), the present model of planetary health literacy is based on the four competencies related to the process of accessing, understanding, appraising, and applying health-related information from the integrated model of health literacy by Sørensen et al. (11). The definition by Sørensen et al. in turn is based on a systematic literature review and content analysis including 17 definitions of health literacy and 12 conceptual models (11). The four competencies are transferable and essential to the conceptual model of planetary health literacy and are also reflected by the definition of health literacy provided by the WHO Health Promotion Glossary of Terms 2021 (11, 25). Thus, in terms of specific competencies, planetary health literacy is a specification of health literacy in the context of planetary health. To make the four core competencies more concrete, we zoom into the core of the process and specify the knowledge relevant for planetary health at the individual level. “Access” refers to the ability to retrieve particular types or stocks of knowledge regarding the integrated planetary health perspective on the interconnectedness of human health, the state of the natural systems, and human activities for planetary health using different information sources, e.g., internet, media, expert opinions etc.. “Understand” refers to the ability to process and make sense of this information on the interconnectedness between human health, human activities, and the state of the natural systems. “Appraise” refers to the ability to filter, interpret, and evaluate information on the diverse relationships between human activities, human health, and the state of the natural systems. Finally, “apply” refers to the ability to make informed decisions and transfer these into specific ways of acting regarding human activities or human health in the context of the state of the natural systems. These four competencies are connected in a cyclical fashion as shown in Figure 1. In reality, deviations from this cycle occur. For instance, a particular stock of knowledge may be re-appraised after its application.

### 2.2. A life course and transgenerational approach—At the individual, societal, and global level

Planetary health literacy encompasses both a life course and a transgenerational approach and may thereby act across multiple current and future generations (26, 27). The process of accessing, understanding, appraising, and applying knowledge and expertise on planetary health develops across the life course of individuals. Regarding human health at the individual level, planetary health literacy may contribute to physical and mental wellbeing during the life course by disease prevention and health promotion. Furthermore, planetary health literacy entails a transgenerational approach regarding improvements in public health across multiple current and future generations through health co-benefitsof sustainable and transformative actions. For example, the (planetary health literate) decisions and activities of current generations affect ecosystem health, which in turn has effects on future generations (26). Thus, planetary health literacy of individuals and populations of current generations is not only of relevance for their own health, but affects the health of future generations through the interconnectedness of human activities and ecosystem health. Furthermore, the conceptual model of planetary health literacy pursues a whole-of-society perspective that targets individuals of all age groups (irrespective of their personal and professional backgrounds).

From a global health perspective, planetary health literacy may contribute to wellbeing at the population level. By protecting Earth's natural systems, climate change and the erosion of natural resources can be slowed down. Thus, planetary health literacy goes beyond the individual health level across the life course, and includes the societal and global level across multiple generations.

Based on the conceptual model and on the definition of planetary health by Whitmee et al. (5) we propose the following definition of planetary health literacy:

*Planetary health literacy can be defined as the knowledge and competencies of accessing, understanding, appraising, and applying information in order to make judgements and take decisions regarding planetary health, across societies and for health-promoting, sustainable, and transformative actions*.

*Planetary health literate individuals and societies are enabled to sustain and promote their own health, population health, and the planet's health. They are able to adopt a more holistic understanding of their health embedded in natural systems they are living in. Based on their knowledge and attitude, they take decisions that reflect and foster the interconnectedness of human health and well-being with the state of the natural systems and related areas of nature-society interactions*.

### 2.3. Planetary health literacy as goal of education for planetary health

Our concept of planetary health literacy should be considered and integrated as a goal of education for planetary health, and could be relevant to educational programs focusing on planetary health that use for example the Planetary Health Education Framework (28, 29) or other existing principles and concepts for planetary health education (30, 31). Teaching should go beyond the sole information provision and actually equip individuals with competencies to transfer lessons learned from one example to another. A lesson on healthy and sustainable nutrition should always focus on making this concrete learning transferrable to another decision situation. For example, when teaching about the choice of (healthy and sustainable) vegetables, it should enable people to access, understand, appraise, and apply the understanding of a better consumption choice when purchasing other goods as well. Especially the facet “appraise” as the ability to individually weigh facts against another, e.g., personal preferences against better outcomes for individual and global health, needs to be addressed to help people make better, but still acceptable choices for themselves and the planet. In this context, rational, evidence-based knowledge should be contextualized as Western knowledge and enhanced by insights and practices from Indigenous People, which have for instance been described as being characterized by a posture of humility toward our planet Earth (20).

The addressees of planetary health education need to acquire a wider understanding and necessary skills to be enabled to make health-related decisions for themselves and social and community networks while considering the natural systems as well. Furthermore, planetary health education shall seek to increase motivation in learners for sustainable practices and increase learners' confidence, that they can make a meaningful contribution to transformative change on a larger scale. Through transformative competences that enable transformative actions (10), these skills will promote changing personal behaviors and human activities as well as social actions influencing others (32). Health education with high levels of planetary health literacy as desired outcome enables people to make informed decisions to improve planetary health by enhancing their ability to seek, critically evaluate, and use health information while at the same time adopting a more holistic vision of their health embedded in natural systems they are living in (33). Generic, transferable skills can be developed to equip people to make a range of more autonomous decisions relating to their and the planet's health and to adapt to changing circumstances (34).

Educational interventions need to deal with the enormous complexity of nowadays individual and collective choices and the implications of these complex choices. Further, educational interventions need to promote systems thinking as a crucial foundation for planetary health literacy. To foster transformative action, it is important to enable learners to balance the presumed ideal individuals' choices with the limited rooms for action they have. It is equally important to build up resilience in learners and prevent frustration by dissonance between the knowledge about necessary changes on a political level and the observed political action.

In order to take a life course and transgenerational approach, lifelong learning should be an integral aspect of both education for planetary health and planetary health literacy. This is especially important because planetary health literacy should be strengthened in the whole of society. Through age-appropriate education, children of different ages can be engaged in numerous ways to interact with nature and develop a deep connection.

### 2.4. Zoom out: The potential and opportunities of planetary health literacy

When zooming out of the process of planetary health literacy away from the individual level toward a more holistic view on the planetary level, we can see the potential and relevance of education focusing on planetary health literacy as its outcome. Education allows individuals to make sense of their environment and thus enables them to make changes (35). Education that fosters planetary health literacy may enable individuals of all ages and in all regions of the world to promote, protect and improve their own health, population health, and the planet's health. Integrating planetary health literacy in education for planetary health can address the three main challenges identified by Whitmee et al. to achieving planetary health: imagination, knowledge, and implementation challenges (5).

Planetary health literacy enables individuals to make informed decisions to improve planetary health—under consideration of the deep interconnectedness of human health and the natural systems making up our planet. For human health, informed decisions mainly affect disease prevention and health promotion. For the natural systems, informed decisions are related to sustainable environment protection and the precautionary principle.

Depending on the influence and societal role of a planetary health literate individual (e.g., corporate or political leaders or decision-makers), decisions of this individual may positively contribute to planetary health of current and future societies. Planetary health literacy may thus serve as a concept with enormous potential to address the urgency of the planetary state of emergency we are currently facing—both from a bottom-up perspective and from a top-down perspective—for individual behavior choices and for structural and societal changes. More importantly, planetary health literate societies (i.e., a society that consists of a high proportion of planetary health literate individuals), may positively impact upon planetary health (including public and global health) through collective action, regarding for example climate change mitigation, and may thus contribute to reaching positive social tipping points toward a civilizational transformation for global sustainability (9). Furthermore, planetary health literate societies may contribute to the achievement of the sustainable development goals and of subsequent global agendas.

In addition, the concept of planetary health literacy is in line with the principles of the Geneva Charter for Wellbeing that aims to catalyze the role of health promotion by “ensuring that people and communities are enabled to take control of their health and lead fulfilling lives with a sense of meaning and purpose, in harmony with Nature, through education, culturally relevant health literacy, meaningful empowerment and engagement” (18). Hereby, education should include education for planetary health, and health literacy should be complemented by planetary health literacy. Planetary health literacy may thus contribute to the “transition to more sustainable, equitable societies” with “equitable health now and for future generations without breaching ecological limits” (18). It may thereby contribute to “harmonious relations between humans and nature and center indigenous knowledge and leadership” (18).

### 2.5. Examples of planetary health literacy

In order to illustrate the scope of planetary health literacy, we will explain the application of planetary health literacy for the thematic area of healthy and sustainable food in more detail. The EAT-Lancet Commission on healthy diets from sustainable food systems concludes that “food is the single strongest lever to optimize human health and environmental sustainability on Earth” (36, 37). In order to achieve the overarching goal to provide nearly 10 billion people by 2050 with healthy food within the planet's boundaries, the Commission defines two scientific targets for healthy diets and sustainable food production. The first target addresses healthy diets, i.e., “diets [that] have an optimal caloric intake and consist largely of a diversity of plant-based foods, low amounts of animal-source foods, contain unsaturated rather than saturated fats, and limited amounts of refined grains, highly processed foods and added sugars” (37). Globally, a shift from current diets toward healthy diets includes “doubling in the consumption of healthy foods such as fruits, vegetables, legumes and nuts, and a >50% reduction in global consumption of less healthy foods such as added sugars and red meat” (37). The second target addresses sustainable food production and the Commission “proposes boundaries that global food production should stay within to decrease the risk of irreversible and potentially catastrophic shifts in the Earth system” (36).

In the context of healthy and sustainable diets, the proposed concept for planetary health literacy describes the process of (1) the ability to access information on the interconnectedness of eating behavior, human health, and the state of the natural systems regarding a healthy and sustainable diet (e.g., reduction of meat consumption, consumption of local and seasonal fruit and vegetables) for planetary health; (2) the ability to understand this information and its implications for personal choices regarding purchase and consumption of food; (3) the ability to interpret and evaluate this information weighing personal preferences, cost implications, cultural imprinting, and influence on the natural systems, and to (4) draw a planetary health literate and informed decision regarding purchase and consumption of food. Thus, it is the process in which planetary health related information is processed by individuals and societal groups, turning information into knowledge, expertise, and the ability to act.

Planetary health literate decisions regarding healthy and sustainable food will primarily be taken by consumers who decide which foods they buy and eat. These decisions will impact upon the consumers' personal food consumption—but may also influence the food consumption of other surrounding individuals (e.g., family members, friends). A body of planetary health literate people acting as a *group* holds the potential to transform food environments from a bottom-up perspective. However, it is restricted to *individual* human activities in terms of behaviors regarding consumption of food, which is strongly influenced by commercial determinants such as the social and economic environment in which these individuals live and work. The availability, cultural desirability, and prices of products influence choices.

But planetary health literate decisions regarding healthy and sustainable foods may also be taken by individuals who act as decision-makers at the level of food production and supply. This may lead to structural changes that enable healthy and sustainable food consumption at a larger scale that goes beyond the individual level and impacts upon public or even global public health. Therefore, far-reaching planetary health literate decisions regarding the provision of healthy and sustainable food (e.g., by the decision-makers within an influential multinational food companies, or decision-makers responsible for food supply in canteens) may address both individuals and populations at the regional and at the global level, and may impact upon the life course of individuals of both current and future generations.

Table 3 shows several examples of application of the four core competencies of planetary health literacy for concrete examples in the thematic area of healthy and sustainable food and in other thematic areas that are relevant for planetary health.

**Table 3:** Examples of thematic areas and of their application regarding the four core competencies of planetary health literacy.

	**Access/obtain information regarding planetary health**	**Understand information regarding planetary health**	**Appraise/judge information regarding planetary health**	**Apply/use information relevant to planetary health**
Overarching contents regarding planetary health literacy	Ability to access information on the interconnectedness of human health, the state of the natural systems, and human activities for planetary health.	Ability to understand information on the interconnectedness between human health, human activities, and the state of the natural systems.	Ability to interpret and evaluate information on the diverse relationships between human activities, human health, and the state of the natural systems.	Ability to make informed decisions regarding human activities or human health in the context of the state of the natural systems.
*Example 1) Active transport for planetary health*	*I am able to access a smartphone app that informs me about the benefits of going to the supermarket by bicycle (instead of going by car) for my personal health (e.g., in terms of calories burned, meeting physical activity recommendations, the associated short and long term positive effects for mental and physical health), and for ecosystem health (e.g., in terms of reduced emissions)*.	*I am able to understand the information provided by the smartphone app and I can derive the meaning of this information regarding the benefits for my own health, ecosystem health and thus for planetary health by going to the supermarket by bicycle instead of taking the car*.	*I am able to interpret the information provided by the smartphone app. This enables me to evaluate the information and to judge the pros and cons of going to the supermarket by bicycle or by car – even under changing environmental conditions (such as heat waves etc.)*.	*I am able to make an informed decision and to draw a planetary health literate conclusion regarding the decision to go by bicycle or by car – even under changing environmental conditions (such as heat waves etc.)*.
*Example 2) Healthy and sustainable diet at the individual level*	*I am able to access information that informs me on nutritional value (calories, vitamins etc.) and impact on ecosystem health (e.g. emissions because of transportation, waste of natural resources because of crop growing, etc.). I am able to access information on the nutritional value and benefits of an avocado provided in the vegetable section. At the same time I can access information on ecosystem impacts of this avocado (e.g. origin, transport costs and emissions, resources needed to grow (high need of water))*.	*I am able to easily understand the information provided and I can derive the meaning of this information regarding the benefits for my own health, ecosystem health by choosing certain fruit or vegetables, and dismissing others*. *I am able to understand which values the consumption has for my personal health (calories, unsaturated fats, etc.) and what the purchase implies regarding planetary health*.	*I am able to interpret the information provided. It enables me to evaluate the information and helps to weigh the pro and cons and thus decide which vegetable or fruit to buy. I am able to appraise this information and weigh the benefits eating an avocado regarding my personal preferences and health against the harm on the ecosystem*.	*I am able to make an informed decision and draw a planetary health literate decision regarding which fruit or vegetable to buy*. *I am able to make an informed decision if I want to buy the avocado, because I like to eat avocado and it is beneficial for my health or if I choose not to buy it, due to the impact on the ecosystem*.
*Examples 3)a-b) Healthy and sustainable diet – individual planetary health literacy for structural changes*	*As one of the responsible persons in school management of a primary school, I am able to access information regarding the benefits of the planetary health diet for both human and ecosystem health during a training session on sustainable foods in school canteens*.	*I am able to understand the information provided during the training session regarding the (short and long term) health benefits for schoolchildren and for ecosystem health that are associated with regular healthy and sustainable diet*.	*I am able to interpret the information provided in the training session. This enables me to critically evaluate the information and to judge the pros and cons of healthy and sustainable food offered in the school canteen*.	*I am able to make an informed decision and to draw a planetary health literate conclusion by changing the supply of food in the school canteen toward a healthy and sustainable planetary health diet*.
	*As the head of the Ministry of Food and Agriculture, I am able to access information regarding the benefits of the planetary health diet for both human and ecosystem health in the current reports on planetary health from several scientific advisory councils*.	*I am able to understand the information provided in the report, especially the recommendations for policy makers regarding the urgent need for a healthy and sustainable food system*.	*I am able to interpret the information provided in the report. This enables me to critically evaluate the information and to judge the pros (and cons) of a healthy and sustainable food system for population health and the state of the natural systems*.	*I am able to make informed decisions and to draw planetary health literate conclusions by doing everything in my power and in my function to overcome existing barriers*.
*Example 4) Prevention of heat wave associated health risks*	*As a person at risk for health complications due to heat waves, I am able to access a health professional that informs me about the potential health effects of heat waves and about preventive measures*.	*I am able to understand the information provided by the health professional and I can derive the meaning of this information regarding my own health risk and potential preventive measures in case of heat waves*.	*I am able to interpret the information provided by the health professional. This enables me to evaluate the information regarding my own heat wave related health risks and to judge the feasibility of preventive measures in case of heat waves*.	*I am able to make an informed decision in case of a heat wave, regarding measures to protect me from negative heat-associated effects on my health*.

### 2.6. Potential barriers and challenges

A broader implementation of the proposed conceptual model of planetary health literacy implies several potential barriers and challenges that need to be addressed. First of all, acceptance of the proposed concept by the scientific community, by educators from all levels of education as well as practitioners from a wide range of disciplines is crucial. Second, the proposed theoretical model needs to be applied in practice of education for planetary health. Thereby, the scope and diversity of planetary health literacy may help to improve education for planetary health. However, the adaptation and application of the planetary health literacy model to different education systems (both within and between countries) may be challenging. Cultural contexts regarding customs, traditions, status symbols etc. need to be considered carefully to allow for sustainable change. Furthermore, it has to be considered that the model proposed here is based on Western rationality, rooted in what has been critiqued as evidentialism and a transactional perspective of changing the world through evidence-based education. The fact that all authors are part of, and trained within, this knowledge culture, could be seen to constrain the extent to which other forms of knowledge have informed the proposed model of planetary health literacy. This limitation could potentially inform the design of future work.

Contextual and structural barriers including for example the influence of large multinational food companies, economic incentives that foster unhealthy and unsustainable behaviors of individuals, or traffic systems that make active transportation difficult, dangerous, or even impossible represent a challenge for transformative actions of planetary health literate individuals and societies. Another challenge refers to the provision and accessibility of information regarding planetary health. The quality of the information provided, the accessibility, and the individual as well as institutional capacities to turn this information into understanding and action need to be ensured.

Furthermore, education for planetary health needs to be provided for individuals of all ages and the corresponding core competencies regarding the process of accessing, understanding, appraising, and applying the information, need to be trained in primary care, kindergarten, primary and secondary schools, vocational trainings, universities, and workplaces. Current and future teachers and health professionals play a key role as “health literacy agents” and need to acquire planetary health literacy for themselves and the corresponding capability to provide planetary health literacy centered education or medical consultation. Specifically, the healthcare system should play a crucial role by integrating planetary health literacy as integral part of education of health professionals and thus enable all health professionals to provide planetary health literacy centered medical consultations and medical care. However, the healthcare system is characterized by increasing profit orientation, high workloads, and a shortage of professionals.

The complexity and the wealth of information regarding planetary health as well as the determinants that influence information environments constitute challenges that need to be addressed. Lessons learnt from the COVID-19 pandemic related “infodemic” [i.e., “too much information including false or misleading information in digital and physical environments during a disease outbreak” (38, 39)] as an important determinant of health, may be applicable to information environments regarding planetary health. Thus, digital (planetary health) literacy is crucial for planetary health literacy and in line with our definition of planetary health literacy refers to the knowledge and competencies of accessing, understanding, appraising, and applying information in digital environments in order to make judgements and take decisions regarding planetary health. But even more crucial is to address the political dimensions of planetary health literacy (39), including the influence and lobby of the oil, food, automobile, pharmaceutical, and other powerful industries through a global governance that targets these new ethical and political challenges.

## 3. Future research needs

Future research needs to empirically validate the conceptual model of planetary health literacy in large scale cohort studies as well as in specific target groups [such as relevant “health literacy agents” e.g.. health professionals (40)]. Here, an inclusive and participatory approach is crucial, and qualitative data collection in a broad range of focus groups in diverse cultural and economic settings is promising. Further, education systems and the barriers on individual and communal level to pedagogically engage with planetary health topics has to be systematically assessed, and structurally transformative education and research policies need to be developed for planetary health literacy. In order to assess planetary health literacy, a set of indicators to quantify planetary health literacy needs to be determined. Furthermore, the association between different levels of planetary health literacy and health outcomes as well as other sustainable, climate-friendly behavior outcomes can be assessed. In addition, participatory development of education formats that enable people to develop planetary health literacy and the assessment of the effects of these formats is crucial. Importantly, different ways of knowing (including diverse perspectives across cultures and contexts), and how they are related to education for planetary health and planetary health literacy need to be assessed. Specifically, addressing Indigenous forms of knowledge on the interplay between the determinants of planetary health (20), education for planetary health (29–31) and planetary health literacy may allow for an understanding of the limits of Western, rational and evidence-based knowledge and may contribute to a deeper understanding of the interconnectedness between humans and nature. Future research should investigate the implications of how equity, justice and power dynamics are addressed in education for planetary health on planetary health literacy in current and future generations.

## 4. Conclusion

This viewpoint provides a conceptual model of planetary health literacy with details on the core competencies of accessing, understanding, appraising, and applying information within knowledge environments regarding planetary health. It allows a holistic planetary health perspective and shows the potential and opportunities of planetary health literacy for the health of both humans and ecosystems. Planetary health literacy encompasses both a life course and a transgenerational approach, at the individual, societal, and global level. Future educational programs focusing on planetary health could integrate the conceptual model of planetary health literacy in order to increase planetary health literacy among individuals (including relevant multipliers) and societies.

## Data availability statement

The original contributions presented in the study are included in the article, further inquiries can be directed to the corresponding author.

## Author contributions

CJ and JS: conceptualization, visualization, and writing (original draft, review, and editing). A-KH and E-MS-S: writing (review and editing). CA: conceptualization, writing (review and editing), and supervision. All authors contributed to the article and approved the submitted version.

## References

[1] United Nations Environment Programme (UNEP). Making Peace With Nature: A Scientific Blueprint to Tackle the Climate, Biodiversity and Pollution Emergencies. Nairobi: UNEP (2021).

[2] Intergovernmental Panel on Climate Change (IPCC). Climate Change 2014: Synthesis Report. Contribution of Working Groups I, II and III to the Fifth Assessment Report of the Intergovernmental Panel on Climate Change. Geneva: IPCC (2014).

[3] Intergovernmental Science-Policy Platform on Biodiversity and Ecosystem Services (IPBES). Global Assessment Report on Biodiversity and Ecosystem Services of the Intergovernmental Science-Policy Platform on Biodiversity and Ecosystem Services. Bonn: IPBES secretariat (2019) 20.

[4] Watts N Adger WN Agnolucci P Blackstock J Byass P Cai W. Health and climate change: policy responses to protect public health. Lancet. (2015) 386:1861–914. 10.1016/S0140-6736(15)60854-6 26111439

[5] Whitmee S Haines A Beyrer C Boltz F Capon AG de Souza Dias BF. Safeguarding human health in the Anthropocene epoch: report of The Rockefeller Foundation-Lancet Commission on planetary health. Lancet. (2015) 386:1973–2028. 10.1016/S0140-6736(15)60901-1 26188744

[6] GBD 2019 Diseases and Injuries Collaborators. Global burden of 369 diseases and injuries in 204 countries and territories, 1990-2019: a systematic analysis for the Global Burden of Disease Study 2019. Lancet. (2020) 396:1204–22. 10.1016/S0140-6736(20)30925-9 33069326 PMC7567026

[7] GBD 2019 Risk Factors Collaborators. Global burden of 87 risk factors in 204 countries and territories, 1990-2019: a systematic analysis for the Global Burden of Disease Study 2019. Lancet. (2020) 396:1223–49. 10.1016/S0140-6736(20)30752-2 33069327 PMC7566194

[8] World Health Organization. Trends in Maternal Mortality 2000 to 2017: Estimates by WHO, UNICEF, UNFPA, World Bank Group and the United Nations Population Division. Geneva: World Health Organization (2019).

[9] Lenton TM Benson S Smith T Ewer T Lanel V Petykowski E.. Operationalising positive tipping points towards global sustainability. Global Sustain. (2022) 5:1–16. 10.1017/sus.2021.30

[10] Schneidewind U. Transformative Literacy: Gesellschaftliche Veränderungsprozesse verstehen und gestalten. GAIA. (2013) 22:82–6. 10.14512/gaia.22.2.5

[11] Sørensen K Van den Broucke S Fullam J Doyle G Pelikan J Slonska Z. Health literacy and public health: a systematic review and integration of definitions and models. BMC Public Health. (2012) 25. 10.1186/1471-2458-12-80 22276600 PMC3292515

[12] Decamps A. Analysis of Determinants of a Measure of Sustainability Literacy. UNESCO (2017).

[13] Orr D. Ecological Literacy. London: Earthscan (2009).

[14] Goleman D. Ecoliterate: How Educators Are Cultivating Emotional, Social, and Ecological Intelligence Somerset. San Francisco, CA: John Wiley & Sons Incorporated (2012).

[15] Hollweg KS TJ Bybee RW Marcinkowski TJ McBeth WC Zoido P. Developing a Framework for Assessing Environmental Literacy. Washington, DC: North American Association for Environmental Education (2011).

[16] Limaye VS Grabow ML Stull VJ Patz JA. Developing a definition of climate and health literacy. Health Aff. (2020) 39:2182–8. 10.1377/hlthaff.2020.01116 33284692 PMC8428792

[17] Finn S O'Fallon L. The emergence of environmental health literacy-from its roots to its future potential. Environ Health Perspect. (2017) 125:495–501. 10.1289/ehp.1409337 26126293 PMC5382009

[18] World Health Organization. Geneva Charter for Well-Being. Geneva: World Health Organization (2021).

[19] Galway LP Parkes MW Allen D Takaro TK. Building interdisciplinary research capacity: a key challenge for ecological approaches in public health. AIMS Public Health. (2016) 3:389–406. 10.3934/publichealth.2016.2.389 29546171 PMC5690363

[20] Redvers N Celidwen Y Schultz C Horn O Githaiga C Vera M. The determinants of planetary health: an Indigenous consensus perspective. Lancet Planet Health. (2022) 6:e156–63. 10.1016/S2542-5196(21)00354-5 35150624

[21] Tu'itahi S Watson H Egan R Parkes MW Hancock T. Waiora: the importance of Indigenous worldviews and spirituality to inspire and inform Planetary Health Promotion in the Anthropocene. Glob Health Promot. (2021) 28:73–82. 10.1177/17579759211062261 34931576 PMC8821976

[22] Nutbeam D. The evolving concept of health literacy. Soc Sci Med. (2008) 67:2072–8. 10.1016/j.socscimed.2008.09.050 18952344

[23] Paasche-Orlow MK Wolf MS. The causal pathways linking health literacy to health outcomes. Am J Health Behav. (2007) 31 Suppl 1:S19–26. 10.5993/AJHB.31.s1.4 17931132

[24] DeWalt DA Broucksou KA Hawk V Brach C Hink A Rudd R. Developing and testing the health literacy universal precautions toolkit. Nurs Outlook. (2011) 59:85–94. 10.1016/j.outlook.2010.12.002 21402204 PMC5091930

[25] World Health Organization. Health Promotion Glossary of Terms 2021. Geneva: WHO (2021).

[26] Andina T. Climate issue: the principle of transgenerational responsibility. Rivista Estetica. (2020) 75:17–32. 10.4000/estetica.7201

[27] Hagemann E Silva DT Davis JA Gibson LY Prescott SL. Developmental Origins of Health and Disease (DOHaD): the importance of life-course and transgenerational approaches. Paediatr Respir Rev. (2021) 40:3–9. 10.1016/j.prrv.2021.05.005 34148804

[28] Guzmán CAF Aguirre AA Astle B Barros E Bayles B Chimbari M. A framework to guide planetary health education. Lancet Planet Health. (2021) 5:e253–5. 10.1016/S2542-5196(21)00110-8 33894134

[29] Guzman CAF Potter T. The Planetary Health Education Framework. Planetary Health Alliance (2021).

[30] Stone SB Myers SS Golden CD. Cross-cutting principles for planetary health education. Lancet Planet Health. (2018) 2:e192–e3. 10.1016/S2542-5196(18)30022-6 29709278

[31] Shaw E Walpole S McLean M Alvarez-Nieto C Barna S Bazin K. AMEE Consensus Statement: planetary health and education for sustainable healthcare. Med Teach. (2021) 43:272–86. 10.1080/0142159X.2020.1860207 33602043

[32] Nutbeam D. Discussion Paper on Promoting, Measuring and Implementing Health Literacy: Implications for Policy and Practice in Non-communicable Disease Prevention and Control. World Health Organization(2017).

[33] Stars I. Health literacy as a challenge for health education. SHS Web Conf. (2018) 40. 10.1051/shsconf/2018400200434737659

[34] Nutbeam D. Health education and health promotion revisited. Health Educ J. (2019) 78:705–9. 10.1177/001789691877021523136306

[35] Cohen AK Syme SL. Education: a missed opportunity for public health intervention. Am J Public Health. (2013) 103:997–1001. 10.2105/AJPH.2012.300993 23597373 PMC3698749

[36] Willett W Rockström J Loken B Springmann M Lang T Vermeulen S. Food in the anthropocene: the EAT-Lancet Commission on healthy diets from sustainable food systems. Lancet. (2019) 393:447–92. 10.1016/S0140-6736(18)31788-4 30660336

[37] EAT-Lancet Commission. EAT-Lancet Commission Summary Report: Healthy Diets From Sustainable Food Systems - Food, Planet, Health.

[38] World Health Organization. Infodemic. Available from: https://www.who.int/health-topics/infodemic#tab=tab_1 (accessed December 1, 2022).

[39] Kickbusch I. Health literacy-politically reloaded. Health Promot Int. (2021) 36:601–4. 10.1093/heapro/daab121 34417606

[40] Walpole SC Barna S Richardson J Rother HA. Sustainable healthcare education: integrating planetary health into clinical education. Lancet Planet Health. (2019) 3:e6–7. 10.1016/S2542-5196(18)30246-8 30654868

[41] IPCC. Summary for Policymakers. Cambridge; New York, NY (2022).

